# Olaparib, a PARP-1 inhibitor, protects retinal cells from ocular hypertension-associated oxidative damage

**DOI:** 10.3389/fcell.2022.925835

**Published:** 2022-08-26

**Authors:** Yuting Yang, Jihong Wu, Wei Lu, Yiqin Dai, Youjia Zhang, Xinghuai Sun

**Affiliations:** ^1^ Department of Ophthalmology and Visual Science, Eye and ENT Hospital, Shanghai Medical College, Fudan University, Shanghai, China; ^2^ NHC Key Laboratory of Myopia, Chinese Academy of Medical Sciences, and Shanghai Key Laboratory of Visual Impairment and Restoration, Fudan University, Shanghai, China; ^3^ State Key Laboratory of Medical Neurobiology and MOE Frontiers Center for Brain Science, Institutes of Brain Science, Fudan University, Shanghai, China

**Keywords:** chronic ocular hypertension, oxidative damage, RGCs, olaparib, mitochondrial-associated endoplasmic reticulum membrane

## Abstract

Glaucoma is the most common cause of irreversible blindness worldwide. Elevated intraocular pressure (IOP) and relative hypoxia in the retina stimulate the production of reactive oxygen species (ROS), which, in turn, puts the retina and optic nerve under chronic oxidative stress. Emerging evidence has shown that oxidative stress can trigger PARP-1 overactivation, mitochondrial-associated endoplasmic reticulum membrane (MAM) dysregulation, and NLRP3 activation. Oxidative damage can trigger inflammasome activation, and NLRP3 is the only inflammasome associated with MAM dysregulation. In addition, multiple transcription factors are located on the MAM. This study aimed to investigate the protective effects and underlying mechanisms of a PARP-1 inhibitor (olaparib) against chronic ocular hypertension-associated retinal cell damage. We also mimicked hypoxic stimulation of a retinal precursor cell line by exposing the cells to 0.2% O_2_ *in vitro*. We discovered that chronic ocular hypertension (COH) induces oxidative damage and MAM dysregulation in the retinal ganglion cells (RGCs). The protein levels of cleaved-PARP and NLRP3 were upregulated in the retinas of the COH rats. Olaparib, a PARP-1 inhibitor, alleviated COH-induced RGC loss, retinal morphological alterations, and photopic negative response amplitude reduction. Olaparib also relieved hypoxic stimulation-induced loss of cell viability and MAM dysregulation. Additionally, some indicators of mitochondrial performance, such as reactive oxygen species accumulation, mitochondrial Ca^2+^ influx, and mitochondrial membrane potential collapse, decreased after olaparib treatment. Olaparib attenuated the hypoxia-induced upregulation of NLRP3 protein levels as well as the phosphorylation of ERK1/2 and histone H2A.X. These results suggest that olaparib protects RGCs from chronic intraocular pressure elevation *in vivo* and alleviates the abnormal MAM dysregulation and mitochondrial dysfunction caused by hypoxia *in vitro*. This protection may be achieved by inhibiting PARP-1 overactivation, NLRP3 upregulation, and phosphorylation of ERK1/2.

## Introduction

Glaucoma is the most frequent cause of irreversible blindness worldwide and is characterized by progressive retinal ganglion cell (RGC) dysfunction and visual field defects (Jonas et al., 2017). Elevated intraocular pressure (IOP) and relative hypoxia in the retina stimulate the production of reactive oxygen species (ROS), which, in turn, puts the retina and optic nerve under chronic stress (Nita and Grzybowski, 2016). The relationship between oxidative stress and glaucoma-associated RGCs’ death has been studied over recent decades (Tezel et al., 2007). 8-Hydroxy-2 deoxyguanosine (8-OHdG) is a classic marker of oxidative damage. Clinical studies have shown a more elevated expression of 8-OHdG in the body fluids of patients with various types of glaucoma is higher than that in healthy controls (Himori et al., 2016; Lanzi et al., 2019; Kondkar et al., 2020). However, glaucoma remains to have a poor prognosis, indicating that further exploration is needed to better understand RGC death in glaucoma. As such, exploring the prevention of other regulated cell death (except apoptosis) provides new therapeutic strategies for neurodegenerative diseases, such as glaucoma (Qin et al., 2022). Elucidating how molecular pathways underlying RGC death are interconnected will provide insights into future therapeutics for retinal degeneration.

The poly (ADP-ribose) polymerase (PARP) enzyme is a group of DNA-dependent polymerases that transfer the ribose of ADP obtained from nicotinamide adenine dinucleotide (NAD^+^) to various proteins to construct poly (ADP-ribose) [PAR] polymerization substances that regulate physiological processes (Ikejima et al., 1990). As the first discovered and most studied protein of the PARP family, PARP-1 has been shown to play a dual role: it can detect and repair DNA damage and serve as a transcription factor involved in chromatin remodeling, transcription, and regulation of inflammatory processes (Rosado et al., 2013; Kadam et al., 2020). When the damage persists without being effectively repaired, PARP-1 remains overactivated, resulting in NAD^+^ depletion, increased mitochondrial membrane permeability, and ultimately PARP-1-dependent cell death (parthanatos) (Fatokun et al., 2014). There is increasing evidence that parthanatos contributes to neuronal cell death in neurodegenerative diseases (Lee et al., 2013; Li et al., 2019). However, the role of PARP-1 in ocular hypertension-associated oxidative damage has rarely been reported.

Various intracellular molecules that regulate cellular physiological and pathophysiological processes, such as Ca^2+^ homeostasis and autophagy, are in mitochondrial-associated endoplasmic reticulum membranes (MAMs). Calcium is a fundamental intracellular messenger that regulates multiple cellular processes, and the function of MAMs as a platform for calcium transfer has been well studied (Ni et al., 2021). MAM dysfunction is associated with various neurodegenerative diseases (Manganelli et al., 2020; Yuan et al., 2020). The distance between the mitochondria and endoplasmic reticulum (ER) is important for maintaining MAM homeostasis; a significant reduction in this distance has been observed in central nervous system degeneration, including Alzheimer’s disease and Parkinson’s disease (Perez-Leanos et al., 2021). However, alterations in MAMs in retinal cells under ocular hypertension-associated oxidative damage and how MAM dysregulation leads to retinal cell death have not been studied.

Olaparib is a PARP inhibitor that inhibits both PARP-1 and PARP-2 activities. Recent studies have shown that olaparib protects neuronal cells by inhibiting inflammasome activation (Sethi et al., 2019; Yao et al., 2021). Both *in vitro* and *in vivo* experiments have shown that olaparib protects photoreceptors in mice with retinitis pigmentosa by inhibiting PARP-1-dependent cell death (Sahaboglu et al., 2016). In addition, olaparib protects retinal cells from chronic hypoxia/reoxygenation injury by activating Nrf2 and inhibiting ROS production (Kovacs et al., 2019). An increasing number of studies have explored the role of the NLRP3 inflammasome in ocular hypertension animal models (Chi et al., 2014; Pronin et al., 2019). The pathologic inflammatory process in these models involved MAMs (Missiroli et al., 2018), and among the inflammasome subfamilies, NLRP3 is the only inflammasome complex associated with MAMs (Zhou et al., 2011). Moreover, several studies have shown that PARP-1 is involved in the regulation of inflammation in various disease models (Hottiger et al., 2010; Narne et al., 2017). A variety of transcription factors and proteins involved in regulating mitochondrial function in neural cells are located on the MAMs rather than on the mitochondrial outer membrane (Su et al., 2020). Studies have shown that multiple transcription factors, such as histones (Huang et al., 2013) and ERK1/2 (D’espessailles et al., 2018) participate in activating the NLRP3 inflammasome.

This study aimed to explore the alterations of MAMs in retinal cells under oxidative stress. It also aimed to discover whether the PARP-1 inhibitor (olaparib) can relieve retinal cells from chronic ocular hypertension-associated oxidative damage and to elucidate the potential mechanisms underlying these protective effects.

## Materials and methods

### Antibodies

The following commercial antibodies (vendor, catalog number, and dilution) were used according to manufacturers’ instructions (Table 1):

**Table 1:** 

Antibody	Vendor	Catalog number	Dilution ratio
Rabbit anti-cleaved-PARP	Cell Signaling Technology	5625S	WB 1:1000
Rabbit anti-NLRP3	Abcam	ab263899	WB 1:1000
Rabbit anti-phospho-p44/42 MAPK (Erk1/2)	Cell Signaling Technology	8544	WB 1:3000
Rabbit anti-p44/42 MAPK (Erk1/2)	Cell Signaling Technology	4695	WB 1:3000
Rabbit anti-phospho-histone H2A.X	Cell Signaling Technology	9718T	WB 1:3000
Rabbit anti-histone H2A.X	Proteintech	10856-1-AP	WB 1:3000
Mouse anti-actin-HRP	Proteintech	HRP-60008	WB 1:5000
Rabbit anti-8-OHdG	Abcam	ab48508	IHC 1:200
Rabbit anti-RBPMS	Thermo Fisher Scientific	PA5-63383	IFA 1:500
Mouse anti-VDAC1/porin	Abcam	ab14734	PLA 1:200
Rabbit anti-ITPR3	Thermo Fisher Scientific	PA5-79544	PLA 1:200

WB: western blotting, IHC: immunochemistry staining, IFA: immunofluorescent staining, PLA: proximity ligation assay.

### Chronic ocular hypertension model

Male Wistar rats (weight 200 ± 20 g, Animal Experiment Management Department of Shanghai Family Planning Research Institute) were used for this chronic ocular hypertension project. The study was approved by the Animal Care Committee of the Eye & ENT Hospital, Shanghai Medical College, Fudan University; all experiments were carried out following the ARVO statement for the use of animals in ophthalmic and vision research. Wistar rats were housed under environmentally controlled conditions (12-h light-dark cycle) with free access to chow and water.

Chronic ocular hypertension was induced as previously described by microbead injection (Sappington et al., 2010; Chen et al., 2011). In brief, in anesthetized rats, the elevation of IOP was induced unilaterally in adult Wistar rats by the anterior chamber injection of 10 µl microbeads (BangsLab, United States, #BM547, diameter 10 µm, 50 mg/ml). The control group received 10 µl of saline in the anterior chamber. Rats received a second injection of microbeads 2 weeks after the first injection. The rats with corneal opacity or signs of inflammation were excluded from further experiments. IOP was measured at 0, 1, 3, and 5 days; and 1, 2, 3, and 4 weeks after the injections in both eyes using a TonoLab tonometer.

### Intravitreal administration of olaparib

Two days after the microbead injection, rats were given olaparib (MedChemExpress, #HY-10162, 400 nM, 2 µl) or sterile PBS (2 µl) intravitreally every week. The rats were anesthetized, and their eyes were visualized using a surgical microscope after topical anesthesia with oxybuprocaine hydrochloride eye rrops (Santen Pharmaceutical Co., Ltd., Osaka, Japan) and pupil dilation with a mixture of tropicamide phenylephrine eye drops (Santen Pharmaceutical Co., Ltd., Osaka, Japan). For each intravitreal injection, the glass micropipette was inserted into the peripheral retina about 1 mm behind the ora serrata. The rats were divided into four groups: control, control + olaparib, microbead, microbead + olaparib (Supplementary Figure S5).

### Histological examination

At designated time points, the eyes were fixed in 4% paraformaldehyde (PFA) and embedded in paraffin. Sections (thickness: 5 μm) of each eye were prepared in a standard manner and stained with Hematoxylin–Eosin (H&E). Morphometric analysis was performed to quantify ocular hypertension injury. The total retinal thickness (from the internal to the outer limiting membrane) and the thickness of the ganglion cell layer (GCL) + inner plexiform layer (IPL) were measured in adjacent areas of the retina within 1 mm of the optic nerve by using ImageJ software.

### 8-Hydroxy-2 deoxyguanosine (8-OHdG) detection

Slides were prepared as previously described. After antigen retrieval and blocking, the slides were incubated overnight with 8-OHdG antibody and afterward incubated with EnVison^TM^+/HRP-rabbit working buffer for 30 min followed by treatment with peroxidase-labeled streptavidin for 20 min at room temperature. The peroxidase activity was developed with 0.25 mg/ml 3,3′-diaminobenzidine tetrahydrochloride in the presence of 0.003% hydrogen peroxide in 0.05 M Tris-buffered saline at pH 7.4. The percentage of 8-OHdG^+^ RGCs was determined by counting the number of 8-OHdG^+^ and DAPI^+^ RGCs under light microscopy (x 20) and calculating the ratio of the 8-OHdG^+^ to the DAPI^+^ RGCs. At least three representative areas were examined in each specimen.

### Transmission electron microscopy

Enucleated eyes were fixed in 4% paraformaldehyde in 0.1M phosphate buffer (PB) for 5 min, after which the anterior chambers and lenses were dissected away from each eyecup. A small region of the eyecup was then removed and placed in 2.5% glutaraldehyde +2% paraformaldehyde in 0.1M PB (pH 7.4) overnight at 4°C. Tissues were postfixed in 1% osmium tetroxide in PB, dehydrated in ethanol, and embedded in Epon epoxy. Sections (60–90 nm) were cut, stained with 50% ethanoic uranyl acetate and Reynold’s lead citrate, and viewed using a Phillips CM120 transmission electron microscope (FEI Company, Hillsborn, OR). The interface length (threshold distance between 20 and 100 nm) (Csordas et al., 2018) and the distance between the mitochondria and the ER were measured using ImageJ software.

### Retinal ganglion cells loss

The analysis of the RGC loss in the inner retina was performed using a specific anti-RBPMS staining. Whole retinas were flat-mounted, cover-slipped, and specific fluorescence in the inner retina was imaged. Individual retinas were sampled randomly at 12 random fields in three regions/four retinal quadrants at the same eccentricities using a ×40 objective lens. The data from at least three animals were averaged for each group.

### Measurement of the photopic negative response

Four weeks after microbead injection, an Espion Diagnosys System (Diagnosys LLC, United States) was used to record the PhNR. After topical corneal anesthesia with oxybuprocaine hydrochloride eye drops, the PhNR was recorded with the Espion system with wire electrodes placed on the corneal surface of the eye. The test eye was the site of the positive electrode; the ground electrode was inserted into the tail, and the reference electrode was inserted under the skin of the scalp. The stimulus frequency was 2 Hz, and blue light with an intensity of 10 cd/m^2^ was presented for 4 ms against a white background. The PhNR amplitude was defined as the difference between the baseline and the peak of the negative wave following the b-wave.

### Cell culture

The R28 retinal precursor cell line was generously offered by Dr. Guotong Xu (Tongji Eye Institute, Tongji University School of Medicine, Shanghai, China) and cultured in low-glucose Dulbecco’s modified Eagle’s medium (Sigma-Aldrich, United States) with 10% fetal bovine serum (Thermo Fisher Scientific, United States), 100 U/mL penicillin and 100 mg/ml streptomycin (Thermo Fisher Scientific, United States) in a 37°C humidified atmosphere containing 5% CO_2_.

### Hypoxic stimulation and administration of olaparib *in vitro*

R28 cells were plated in 6-well plates at a density of 1.5*10^5^ cells/well, the complete medium was aspirated and low-glucose DMEM supplemented with 5% FBS was added, and then cells were exposed to hypoxia condition (O_2_ concentration 0.2%, CO_2_ 5%). Olaparib was diluted with no-serum media and added into the medium for the indicated time.

### Reactive oxygen species assay

Quantitation of intracellular ROS accumulation was performed by fluorescence detection using the fluorescent probe 2′,7′-dichlorofluorescein diacetate (DCFH-DA) (Beyotime Biotechnology, China). R28 cells were subjected to the appropriate treatments and then incubated for 20 min in the dark at 37 °C with 10 µM DCFH-DA solutions. After incubation, the cells were analyzed within 30 min. Fluorescence images were captured using a fluorescence microscope, and the relative fluorescent intensity was analyzed using ImageJ software.

### JC-1 assay

JC-1 kit (Beyotime Biotechnology) was used to detect mitochondrial membrane potential. In brief, after treatment, cells were rinsed with 1x washing buffer once and then incubated with JC-1 reagent for 20 min at 37°C. Cells were then washed with 1x washing buffer three times and read at 488/525 (monomers) and 550/590 nm (aggregates).

### Rhod-2 staining

Rhod-2 (AAT Bioquest, Canada) was used to measure mitochondrial calcium. R28 cells were plated on 96-well black well/clear bottom plates. After hypoxic stimulation, 100 μl 2x Rhod-2 dye was added into the desired wells containing 100 μl culture medium and incubate the dye-loading plates in the cell incubator for 30 min. Replace the dye working solution with HHBS, and fluorescent images were captured via Zeiss microscopy.

### Mito-tacker and ER-tracker staining and quantitative colocalization analysis

After hypoxia treatments, R28 cells were loaded with 500 nM Mito-Tracker Red FM (Invitrogen, United States) and 1000 nM ER-Tracker Blue-White DPX (Invitrogen, United States) at 37°C for 30 min. R28 cells were then quickly rinsed with warm 1x DPBS (Sigma-Aldrich, United States) once and then fixed with 4% PFA for 5 min at room temperature. Images were captured with confocal microscopy with a ×60 oil immersion objective and processed using NIH’s ImageJ software. Colocalization of ER and mitochondrial markers was calculated as Manders’ colocalization coefficient (MCC) using the JACoP plugin in five randomly selected images per condition in each independent experiment. Auto-thresholds were applied for both channels to select pixels for colocalization analysis.

### Proximity ligation assay

PLA was conducted according to manufactory instructions (DUOLINK^®^, Sigma-Aldrich, France). In brief, rabbit anti-ITPR3 and mouse anti-VDAC1 antibodies were used to label the targeted components. Then, secondary antibodies conjugated with oligonucleotides and ligation solution were sequentially added. The amplification solution was then added together with polymerase. The oligonucleotide arm of one of the PLA probes acted as a primer for a rolling-circle amplification (RCA) reaction using the ligated circle as a template, generating a concatemeric (repeated sequence) product. The fluorescently labeled oligonucleotides would hybridize to the RCA product. The signal was easily visible as a distinct fluorescent spot and analyzed by confocal microscopy (Leica, Germany). A minimum of five images were taken per sample, and three independent series were performed for each treatment. The PLA signal (PLA puncta numbers per cell) was analyzed using the ImageJ software.

### Western blot analyses

Retina or cell lysates were prepared by incubating tissue or cell pellets with ice-cold 1x lysis buffer (Beyotime Biotechnology, China) supplemented with cOmplete^TM^ proteinase inhibitor cocktail (Roche, Switzerland) and phosphatase inhibitor cocktail (Roche, Switzerland) on ice for 20 min. Lysates were then centrifuged at 12,000 g at 4°C for 15 min to remove the insoluble fractions. The supernatant was then mixed with ×5 sample loading buffer (Beyotime Biotechnology, China) and boiled for 7 min. Denatured proteins were separated by SDS-PAGE using 4–20% SurePAGE, Bis-Tris precast protein gels (GenScript, China) and transferred to Immun-Blot^®^ PVDF membranes (Millipore, United States). Membranes were blocked with 5% non-fat milk or 5% BSA in 1x TBS buffer containing 0.05% Tween 20 (TBST), incubated with primary and secondary antibodies diluted according to manufacturers’ instructions, and washed five times with ×1 TBST. Protein bands were visualized with Immobilon Western Chemilum HRP Substrate (Millipore, United States) on a BioSpectrum imaging system (Ultra-Violet Products, China).

### Statistical analysis

Quantitative data were expressed as mean (±SEM). Unpaired Student’s t-test was performed when comparing two groups. One-way ANOVA or two-way ANOVA with Tukey’s test for multiple comparisons were performed when comparing three or more groups. Statistical differences were considered significant when the *p*-value is less than 0.05.

## Results

### Changes in the intraocular pressure and expression of oxidative damage markers in retinal ganglion cells in a chronic ocular hypertension rat model

**FIGURE 1:**
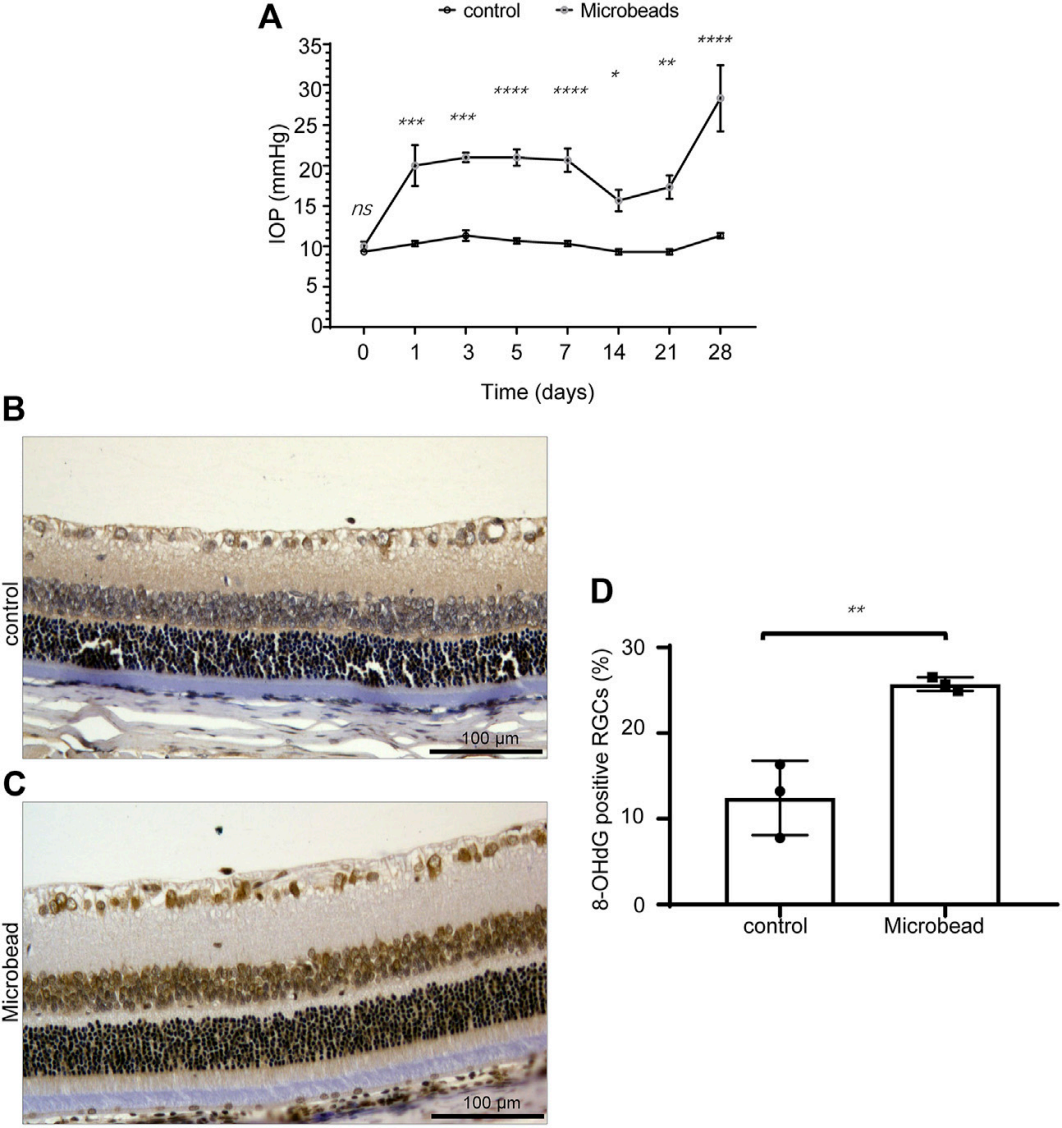
Chronic ocular hypertension-induced oxidative damage in RGCs. **(A)** Compared to the control group, the IOP of the microbead group was significantly increased. **(B,C)** the 8-OHdG^+^ RGCs were significantly increased in the microbead group compared to the control group. Scale bar, 100 µm. **(D)** The *p*-values were determined using an unpaired Student’s t-test for two-group comparisons. ^∗^ *p* < 0.05, ^∗∗^ *p* < 0.01, ^∗∗∗^ *p* < 0.001, and ^∗∗∗∗^ *p* < 0.0001.

After the injection of magnetic beads, the IOP of the COH rats (microbead) increased steadily compared to that of the control rats (control) (Figure 1A). We compared the percentages of 8-OHdG^+^ RGCs in retinal sections from the COH and control rats. The results showed that the 8-OHdG^+^ RGCs were significantly increased in the microbead group compared to the control group (12.4 ± 2.5% in the control group and 25.7 ± 0.5% in the microbead group, *n* = 3, **p = 0.0064) (Figures 1B–D).

### Alterations in mitochondria-associated endoplasmic reticulum membranes in the retinal ganglion cells of chronic ocular hypertension rats

**FIGURE 2:**
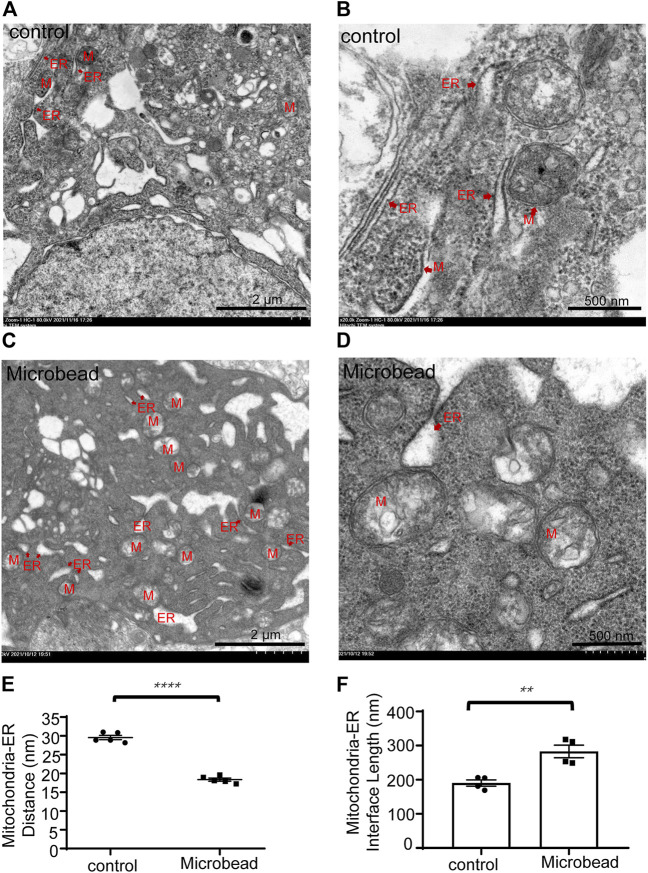
Chronic ocular hypertension injury decreased the distance but increased the interface length between the mitochondria and ER in RGCs. **(A–D)** Representative TEM images of the control group **(A,B)** and the microbead group **(C,D)**. M, mitochondria, ER, endoplasmic reticulum. Scale bar in **(A)** and **(C)**, 2 µm. Scale bar in **(B** and **D)**, 500 nm. **(E,F)** The *p*-values were determined using an unpaired Student’s t-test for two-group comparisons. ^∗∗^ *p* < 0.01 and ^∗∗∗∗^ *p* < 0.0001.

Transmission electron microscope (TEM) images showed that the distance between mitochondria and ER of RGCs in the microbead group (18.35 ± 0.93 nm) was smaller than that of the control group (29.54 ± 1.29 nm) (****P ˂ 0.0001) (Figure 2E) while the interface length between the two organelles was larger in the microbead group (282.85 ± 18.3 nm) than that of the control group (190.39 ± 9.1 nm) (Figure 2F) (**p = 0.004), suggesting that chronic ocular hypertension induces abnormal MAMs in RGCs (Figures 2A–F).

## Chronic ocular hypertension increases cleaved-PARP and NLRP3 protein levels in the retina

**FIGURE 3:**
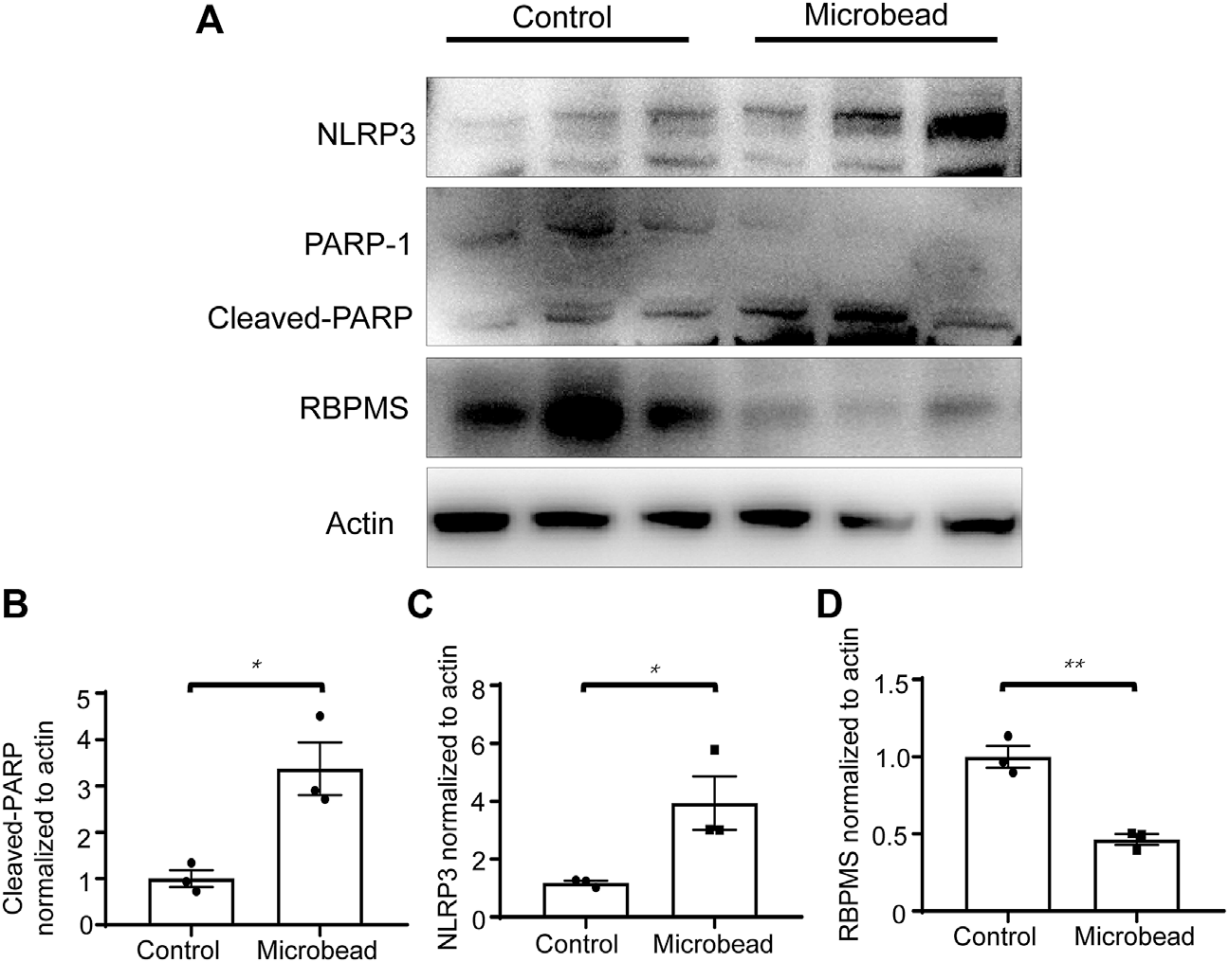
Chronic ocular hypertension decreased RBPMS and increased cleaved-PARP and NLRP3 expression. **(A)** Protein levels of RBPMS, cleaved-PARP, and NLRP3 were analyzed 28 days post-microbead injection. **(B–D)** RBPMS, cleaved-PARP, NLRP3, and actin bands were analyzed via densitometry using the ImageJ software, and the RBPMS/actin, cleaved-PARP/actin, NLRP3/actin ratios were quantified. The *p*-values were determined using an unpaired Student’s t-test for two-group comparisons. ^∗^ *p* < 0.05 and^∗∗^ *p* < 0.01.

Western blotting showed that chronic ocular hypertension reduced the expression level of RNA-binding proteins with multiple splicing (RBPMS) (46.4 ± 0.06% compared to the control group) (**p = 0.0024). Western blotting also showed that compared with the control group, the protein levels of cleaved-PARP and NLRP3 increased 3.38-fold and 3.34-fold, respectively, in the microbead group. (*p = 0.0163 and *p = 0.0406) (Figures 3A–D).

## PARP-1 inhibitor protects retinal ganglion cells from ocular hypertension-associated injury

### Olaparib alleviates chronic ocular hypertension-induced RGC loss

**FIGURE 4:**
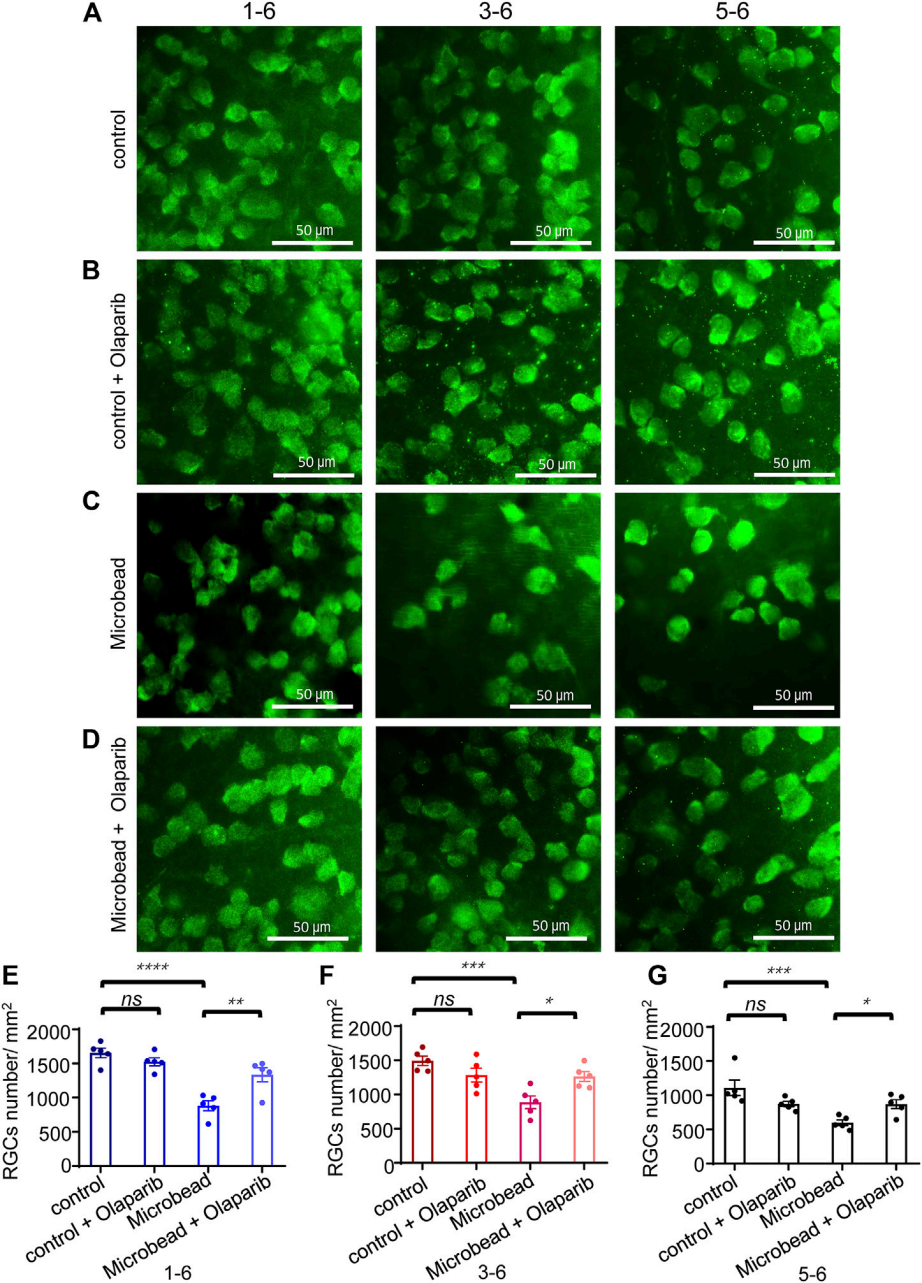
Olaparib alleviates chronic ocular hypertension-induced RGC loss. **(A–D)** Immunofluorescence staining with RBPMS of whole-mount retinas of the control group, control + olaparib group, microbead group, and microbead + olaparib group, respectively. **(E–G)** The bar chart shows the density of surviving RGCs from the four groups. The *p*-values were determined using one-way ANOVA with Tukey’s test for multiple comparisons. Scale bar, 50 μm ^∗^ *p* < 0.05, ^∗∗^ *p* < 0.01, ^∗∗∗^ *p* < 0.001, and ^∗∗∗∗^ *p* < 0.0001.

Retinas were extracted and quantified in the surviving RGCs 28 days after microbead injection (Figures 4A–G). The density of RBPMS^+^ RGCs in eyes with chronic ocular hypertension injury (microbead group, central area, 881.25 ± 73.6/mm^2^, peri-central area, 884.2 ± 92.8/mm^2^, and peripheral area, 597.8 ± 41.1/mm^2^) decreased significantly compared with the control eyes (central area, 1653.35 ± 70/mm^2^, peri-central area, 1492.8 ± 68.3/mm^2^, and peripheral area, 1107.2 ± 112.8/mm^2^). Olaparib injection alone did not cause significant alterations in RGC density (control + olaparib group, central area, 1524.3 ± 58/mm^2^, peri-central area, 1282.5 ± 102.3/mm^2^, and peripheral area, 870.2 ± 38.8/mm^2^) but significantly alleviated chronic ocular hypertension-induced RGC loss (microbead + olaparib group, central area, 1334.84 ± 104.7/mm^2^, peri-central area, 1262.8 ± 72.5/mm^2^, and peripheral area, 868.0 ± 65.7/mm^2^) (Figures 4A–G).

### Olaparib relieves chronic ocular hypertension-induced retinal thickness damage

**FIGURE 5:**
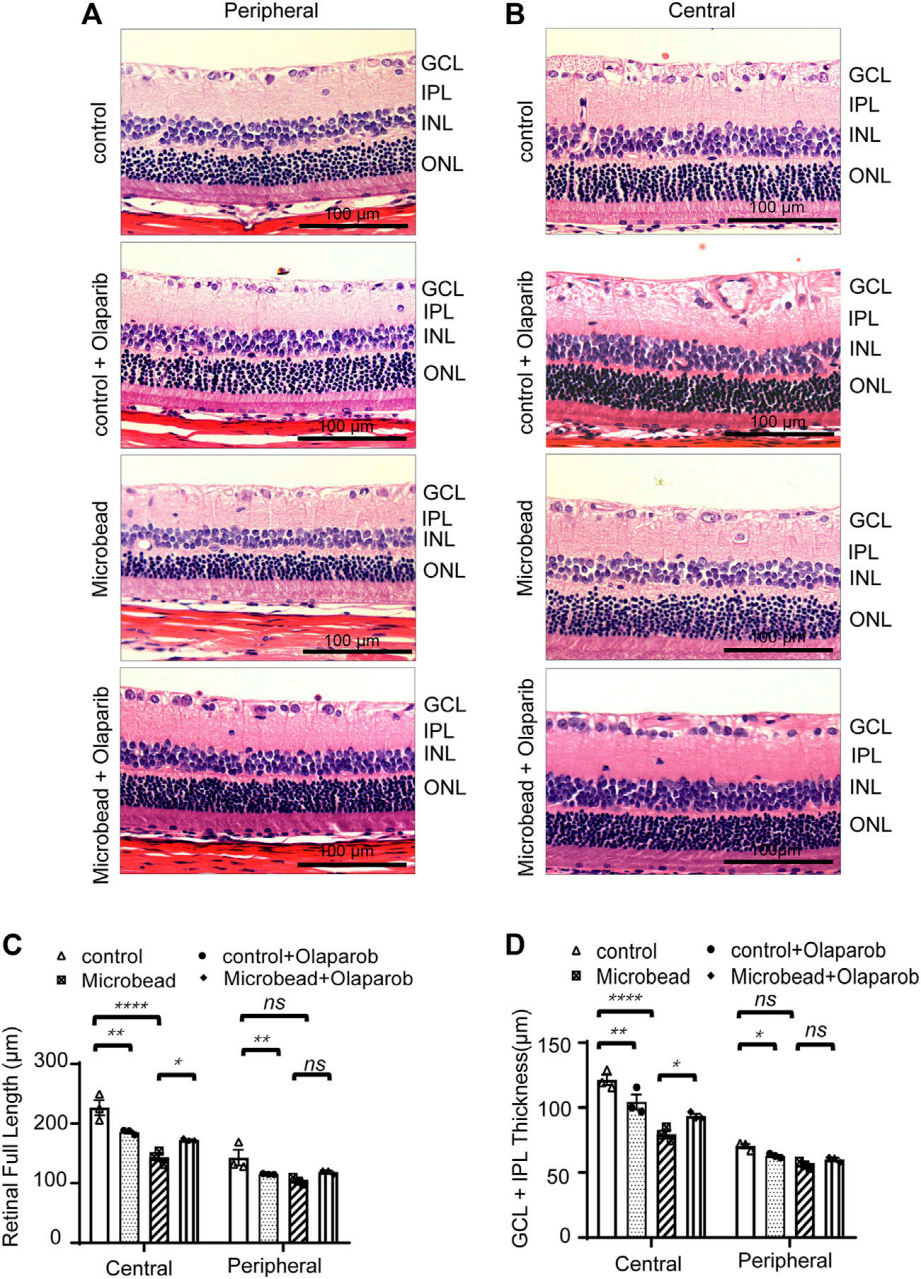
Olaparib relieves chronic ocular hypertension-induced retinal damage as reflected by changes in retinal thickness. H&E staining of retinal slices in the central area **(A)** and peripheral area **(B)** (magnification ×20, scale bar, 100 μm). **(C–D)** Quantitative analysis of retinal (full length) thickness and GCL + IPL retinal thickness. Data are presented as the means ± SEM (n = 3 rats per group). The *p*-values were determined using one-way ANOVA with Tukey’s test for multiple comparisons. ^∗^ *p* < 0.05, ^∗∗^ *p* < 0.01, and ^∗∗∗∗^ *p* < 0.0001.

We next investigated the effect of olaparib on the overall retinal changes using H&E staining. Significant morphological changes were observed in the retina 28 days after chronic ocular hypertension injury compared with the control group (the full retinal thickness was reduced by 36.75% in the central area and 27.15% in the peripheral retina; GCL + IPL retinal thickness was reduced by 34.4% in the central area and 20.6% in the peripheral retina). Administration of olaparib (400 nM) to the microbead group rescued the reduction of the full retinal thickness and the GCL + IPL retinal thickness (*n* = 3, *p < 0.05) (Figures 5A–D).

### Olaparib enhances the PhNR in chronic ocular hypertension rats

**FIGURE 6:**
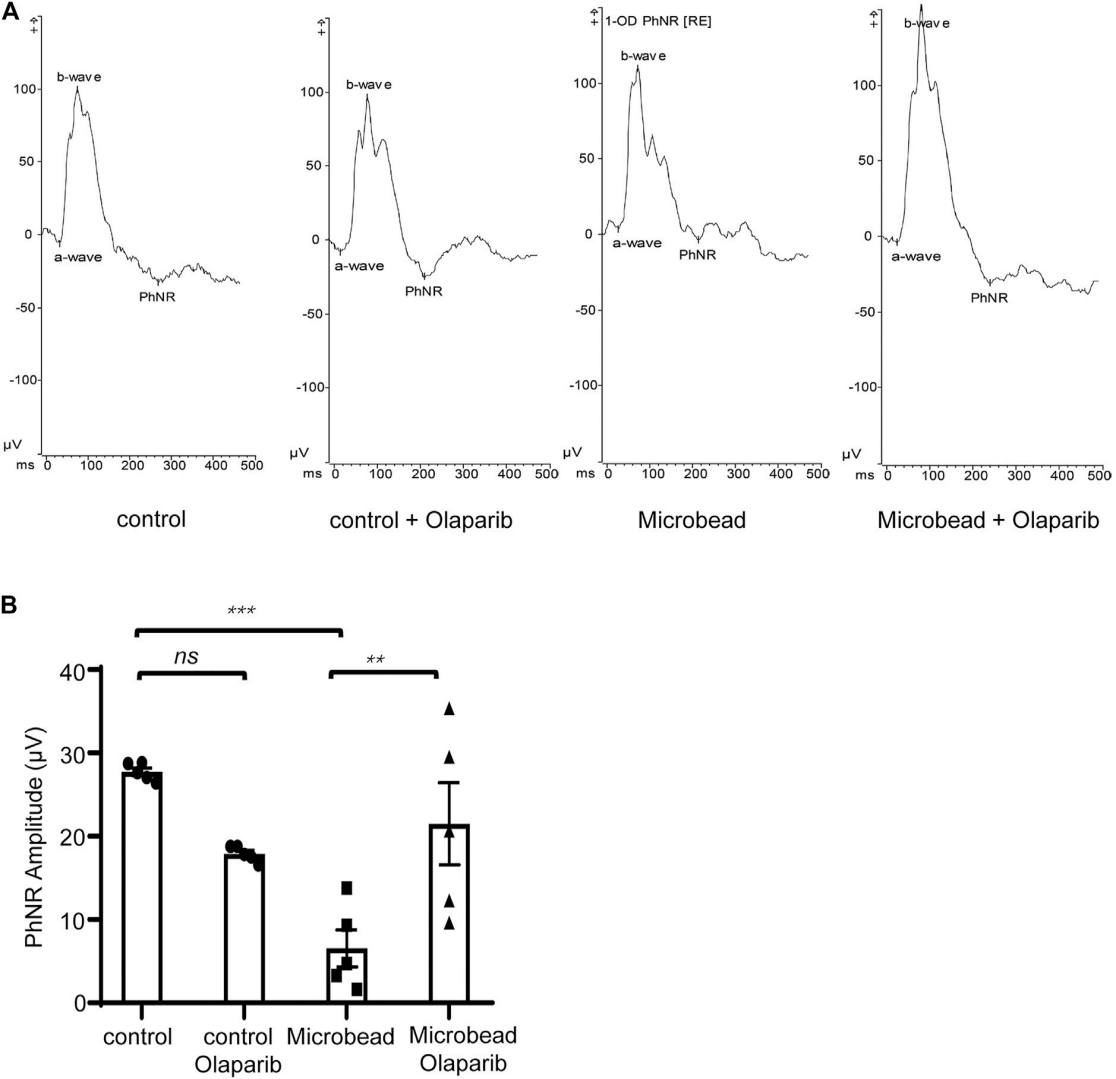
Olaparib enhances the PhNR in the COH rats. **(A)** Typical photopic negative responses (PhNR) in the control, control + olaparib, microbead, and microbead + olaparib group. **(B)** Statistical analysis of PhNR amplitudes in the aforementioned groups. Data are presented as the means ± SEM (*n* = 5 rats per group). The *p*-values were determined using one-way ANOVA with Tukey’s test for multiple comparisons. ^∗∗∗∗^ *p* < 0.0001.

In addition to structural changes, we also detected a protective effect of olaparib on the visual function of RGCs in COH rats. The PhNR amplitude was significantly reduced in the microbead group (6.5 ± 2.21 µV) compared with the control group (27.7 ± 0.47 µV), indicating functional damage to RGCs in rats with chronic ocular hypertension (***p = 0.0001). It is widely accepted that PARP-1 plays an important role in detecting and repairing DNA damage (Kadam et al., 2020). Thus, inhibition of PARP-1 activity in the control group probably caused extra damage. Our results showed that olaparib treatment reduced the PhNR amplitude in the control group (17.9 ± 0.42 µV), although the difference was not statistically significant (*p* = 0.0618). On the other hand, olaparib did significantly improve the PhNR damaged by microbead injection (21.5 ± 4.92 µV) (**p = 0.0039) (Figures 6A,B).

## Mechanisms of olaparib protecting retinal precursor cells from hypoxic damage

To further elucidate the potential mechanisms of the protective effects of olaparib on the RGCs under chronic ocular hypertension-associated oxidative damage, we exposed retinal precursor cells (R28 cells) to hypoxic conditions (0.2% O_2_) *in vitro*.

### Olaparib alleviates hypoxic stimulation-induced cell viability loss, reactive oxygen species accumulation, and mitochondrial membrane potential collapse

**FIGURE 7:**
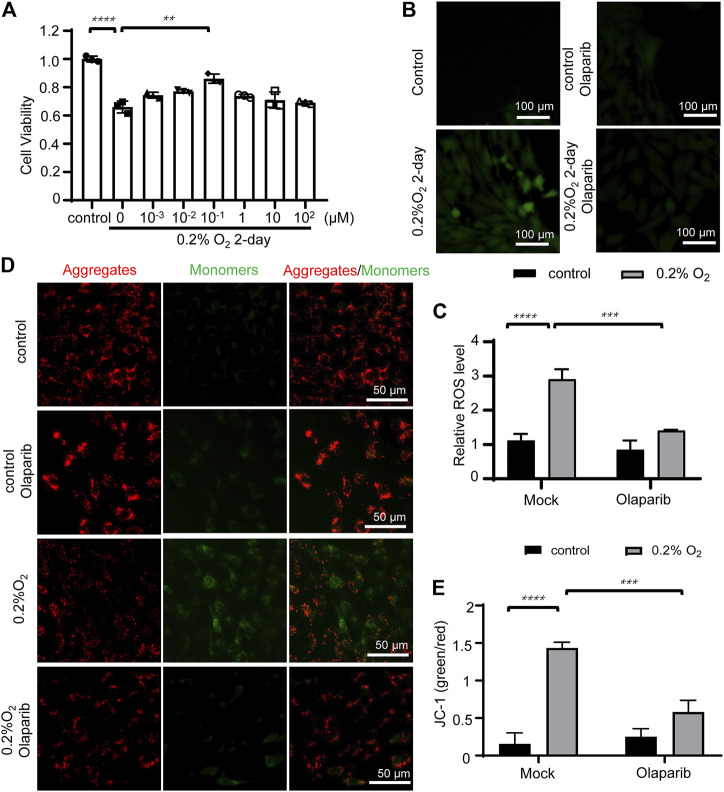
Olaparib alleviates hypoxic stimulation-induced cell viability loss, ROS accumulation, and MMP collapse. **(A)** Olaparib (100 nM) rescued R28 cell viability under hypoxia-induced oxidative stress. The *p*-value was determined using two-way ANOVA with Dunnett’s test for multiple comparisons. Representative images **(B)** and the relative ROS fluorescent intensity **(C)** in the control, control + olaparib, microbead, and microbead + olaparib groups. Data are presented as the means ± SEM. The *p*-values were determined using two-way ANOVA with Sidak’s test for multiple comparisons. Representative images **(D)** and the relative JC-1 green to red fluorescent intensity ratios **(E)** of the aforementioned groups compared to the control group were calculated using the ImageJ software and *p*-values were determined using one-way ANOVA with Tukey’s test for multiple comparisons. ^∗∗^ *p* < 0.01, ^∗∗∗^ *p* < 0.001, and ^∗∗∗∗^ *p* < 0.0001.

Cultured R28 cells were initially exposed to 0.2% O_2_ hypoxia for 12 h. Then serially diluted olaparib (1 nM–100 µM) was added to the culture media, and the cell viability was detected using the CCK-8 kit. The results showed that 100 nM olaparib alleviated hypoxia-induced loss of cell viability; thus this concentration was used in the later experiments (Figure 7A). Compared with the control group, the ROS level in the 0.2% O_2_ hypoxia group increased by 2.5-fold, however, olaparib application reduced this ROS increase (Figures 7B,C). We then detected MMP under normal conditions and hypoxic conditions using the JC-1 kit. Incubation in 0.2% O_2_ induced MMP collapse, which was significantly alleviated by olaparib (Figures 7D,E).

### Olaparib alleviates hypoxia-induced MAM dysregulation

**FIGURE 8:**
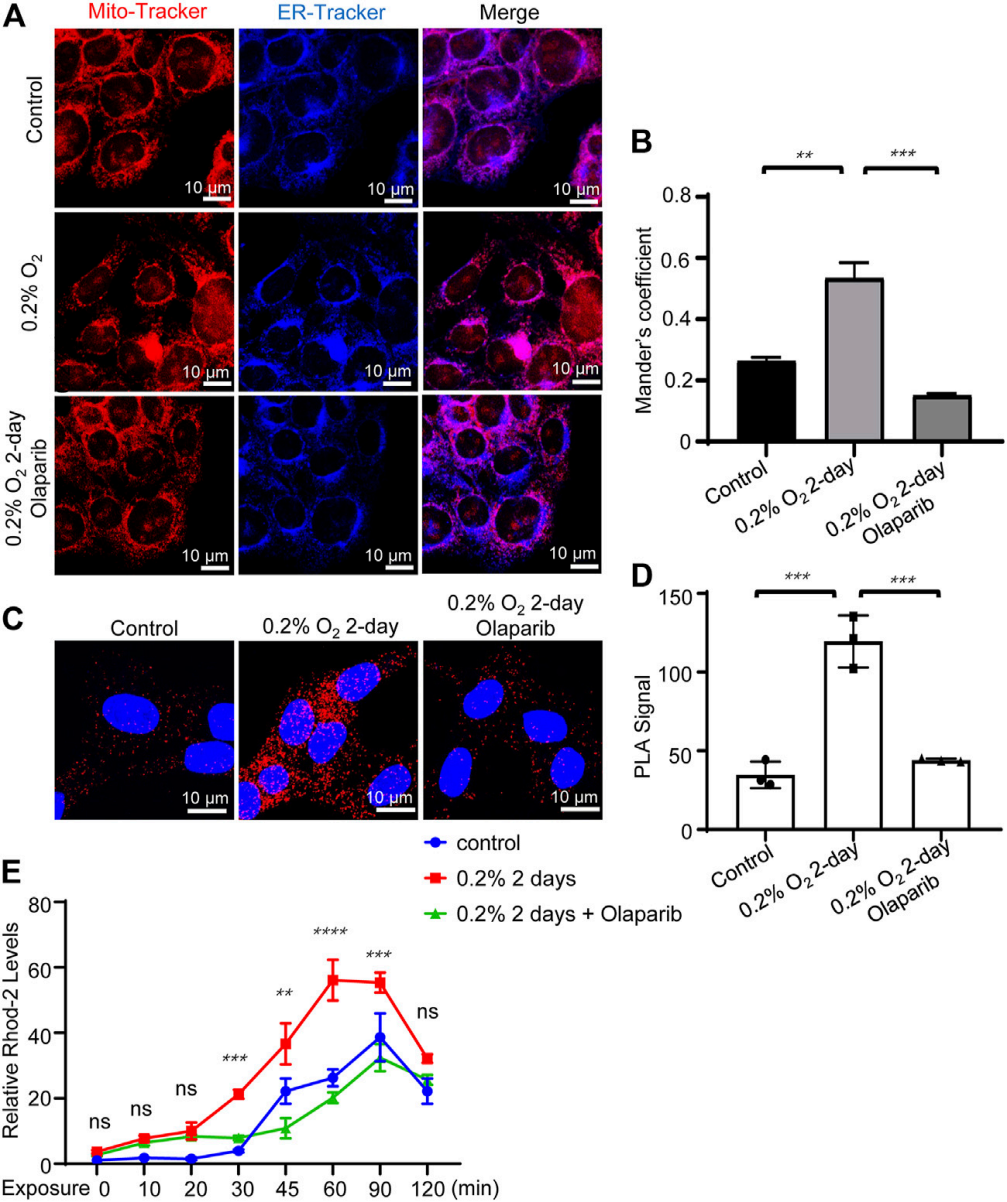
Olaparib alleviates hypoxic stimulation-induced MAMs dysregulation. **(A)** Representative Mito-Tracker and ER-Tracker co-staining fluorescent images for the control, 0.2% O_2_, and 0.2% O_2_ + olaparib groups. Scale bar, 10 μm. **(B)** Colocalization analyses were calculated as Mander’s colocalization coefficients (MCC) using the ImageJ software. **(C)** PLA signals showed that hypoxia (0.2% O_2_) increased MAMs signaling, as evidenced by the increased VDAC1/ITPR3 interactions in R28 cells. PLA signals also showed that olaparib (100 nM) relieved PLA signals under hypoxic stress. Scale bars, 10 μm. **(D)** PLA signals were analyzed using the ImageJ software. **(E)** Rhod-2 staining showed that olaparib relieved hypoxia-induced Ca^2+^ influx. The *p*-values were determined using one-way or two-way ANOVA with Tukey’s test for multiple comparisons. ^∗∗^ *p* < 0.01, ^∗∗∗^ *p* < 0.001, and ^∗∗∗∗^ *p* < 0.0001.

Next, we examined the colocalization of the mitochondria and ER in R28 cells. Cells were exposed to hypoxia for 2 days and then co-stained with Mito-Tracker and ER-Tracker. Olaparib (100 nM) alleviated the increased colocalization of the mitochondria and ER caused by hypoxic stimulation (*p* = 0.0003) (Figures 8A,B). We further used PLA to detect the tethering between mitochondrial VDAC1 and ER ITPR3, in which signals could be detected only if the distance between the mitochondria and ER was <30 nm. The PLA signal alterations were consistent with the MCC analyses, indicating that hypoxia increased MAMs in R28 cells and that olaparib (100 nM) reversed these changes (Figures 8C,D). The mitochondrial calcium concentration was detected via Rhod-2 staining. Hypoxic stimulation increased Ca^2+^ influx at multiple time points compared to that in the control group (30, 45, 60, and 90 min). Olaparib (100 nM) relieved hypoxia-induced Ca^2+^ influx (Figure 8E; Supplementary Figure S2).

#### Olaparib alleviates hypoxia-induced elevation of NLRP3 and phosphorylation of ERK1/2 and histone H2A.X

**FIGURE 9:**
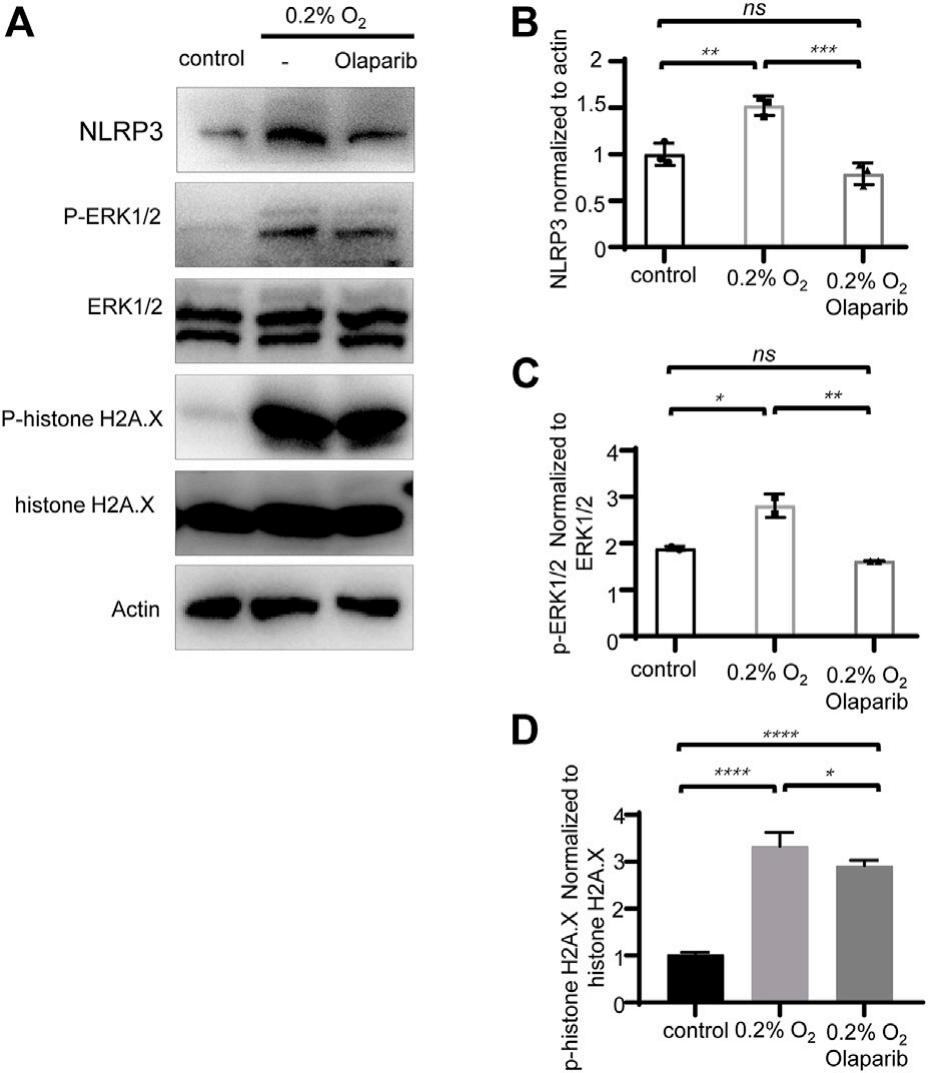
Olaparib alleviates hypoxia-induced elevation of NLRP3, and phosphorylation of ERK1/2 and histone H2A.X. **(A)** Protein levels of NLRP3, P-ERK1/2, ERK1/2, P-histone H2A.X, histone H2A.X, and actin in control, 0.2% O_2_ and 0.2% O_2+_laparib groups. **(B–D)** NLRP3, P-ERK1/2, ERK1/2, P-histone H2A.X, histone H2A.X, and actin bands were analyzed via densitometry using the ImageJ software, and the NLRP3/actin, P-ERK1/2/ERK1/2, and P-histone H2A.X/histone H2A.X ratios were quantified. The *p*-values were determined using one-way ANOVA with Tukey’s test for multiple comparisons. ^∗^ *p* < 0.05, ^∗∗^ *p* < 0.01, ^∗∗∗^ *p* < 0.001, and ^∗∗∗∗^ *p* < 0.0001.

Multiple stimuli, including extracellular ATP, heat shock protein, and double-strand DNA are well-known to trigger NLRP3 inflammasome activation. Our previous results showed an increased NLRP3 protein level in the COH rats’ retina; therefore, we further explored whether hypoxia would stimulate NLRP3 expression in R28 cells. Western blotting showed that compared with the control group, the protein levels of NLRP3 increased 1.52-fold, and olaparib (100 nM) reversed this trend (Figures 9A,B). Our results also showed that hypoxia increased the phosphorylation of ERK1/2 and histone H2A.X in R28 cells, while olaparib alleviated these injurious signaling pathways (Figures 9A,C,D).

## Discussion

PARP-1 is a DNA-dependent polymerase that detects and repairs DNA damage and serves as a transcription factor involved in chromatin remodeling, transcription, and regulation of inflammation (Rosado et al., 2013; Kadam et al., 2020). Although PARP-1 overactivation leads to PARP-1-dependent cell death or parthanatos which is known to contribute to neurodegenerative diseases (Lee et al., 2015; Li et al., 2019), the role of PAPR-1 in ocular hypertension-associated oxidative damage has rarely been reported. Olaparib is an FDA-approved PARP inhibitor that is widely used in ovarian cancer (Dizon, 2017), prostate cancer (Rebello et al., 2021), and pancreatic cancer (Park et al., 2021) treatment. The fact that PARP-1 overactivation triggers lethal pathways provides a rationale for the use of olaparib to protect cells from PARP-1-associated damage. In this study, we discovered that chronic ocular hypertension enhances the expression of cleaved-PARP and NLRP3 and induces MAM dysregulation and mitochondrial dysfunction, whereas vitreous administration of olaparib attenuates COH-induced RGC loss, decreases retinal morphological alterations, and reverses PhNR amplitude reduction. We also tested a lower dose (100 nM) of olaparib as previously reported (Sahaboglu et al., 2016) and found no protective effects on PhNR (Supplementary Figure S4). Further studies are needed to elucidate the effects of higher doses of olaparib on COH-induced RGC injury.

TUNEL staining was used by many studies to detect the neuroprotective effects of olaparib. However, there lies the problem of interpreting the results of this assay, which cannot distinguish among various forms of cell death by itself (Grasl-Kraupp et al., 1995).

Intracellular molecules that regulate physiological and pathologic processes are located on MAMs. MAM dysfunction is associated with various neurodegenerative diseases (Manganelli et al., 2020; Yuan et al., 2020). It has been reported that after stimulation with inducers of the NLRP3 inflammasome, the apoptosis-associated speck-like protein containing a caspase recruitment domain in the mitochondria approaches the NLRP3 inflammasome in the ER, while NAD^+^ supplementation inhibits this trend (Misawa et al., 2013). In addition, previous studies have indicated that sustained Ca^2+^ influx triggers sustained mitochondrial Ca^2+^ overload and destabilization, finally leading to NLRP3 activation (Murakami et al., 2012). In this study, we used the R28 cell line, a retinal precursor cell line, and stimulated them with 0.2% O_2_, to mimic the relative hypoxic conditions and further explore the mechanisms by which olaparib protects retinal cells from ocular hypertension-associated oxidative stress. We found that hypoxia-induced MAM dysregulation and poor mitochondrial performance *in vitro*, accompanied by the upregulation of cleaved-PARP, NLRP3, and phosphorylation of ERK1/2 and histone H2A.X. Meanwhile, olaparib alleviated hypoxia-associated cell viability loss and attenuated hypoxic stimulation-induced MAM dysregulation, mitochondrial dysfunction, NLRP3 upregulation, and ERK1/2 phosphorylation. Given that MAMs play critical roles in regulating mitochondrial function, calcium homeostasis, and inflammation signaling, we propose that MAMs can be targeted for disrupting mitochondrial dysfunction or inflammasome activation and signaling during the neurodegenerative process. Furthermore, it is feasible to assume that the integrity and normal functioning of MAMs are essential for therapeutic intervention. This study had some limitations. R28 is a retinal precursor cell line derived from postnatal day 6 Sprague–Dawley rat retinas and transfected with the 12S E1A gene, resulting in immortalization with no signs of senescence (Seigel, 2014). Thus, R28 differs from primary cells. To better elucidate the protective effects of olaparib, primary RGCs should be used in the future. Additionally, we did not detect alterations in protein levels associated with maintaining the normal function of MAM with or without olaparib. Isolating MAMs fractions and detecting the alterations in the expression profile of the proteins associated with MAM modulation under different conditions may help in elucidating the interrelationships between PARP-1, MAM dysfunction, and lethal signaling.

## Data Availability

The original contributions presented in the study are available in https://www.jianguoyun.com/p/DY-425 vTN4Q9Ym1ChjF7bsEIAA.

## References

[B1] Chen H. Wei X. Cho K. S. Chen G. Sappington R. Calkins D. J. (2011). Optic neuropathy due to microbead-induced elevated intraocular pressure in the mouse. Invest. Ophthalmol. Vis. Sci. 52, 36–44. 10.1167/iovs.09-5115 20702815 PMC3053285

[B2] Chi W. Li F. Chen H. Wang Y. Zhu Y. Yang X. (2014). Caspase-8 promotes NLRP1/NLRP3 inflammasome activation and IL-1β production in acute glaucoma. Proc. Natl. Acad. Sci. U. S. A. 111, 11181–11186. 10.1073/pnas.1402819111 25024200 PMC4121847

[B3] Csordas G. Weaver D. Hajnoczky G. (2018). Endoplasmic reticulum-mitochondrial contactology: structure and signaling functions. Trends Cell Biol. 28, 523–540. 10.1016/j.tcb.2018.02.009 29588129 PMC6005738

[B4] D'espessailles A. Mora Y. A. Fuentes C. Cifuentes M. (2018). Calcium-sensing receptor activates the NLRP3 inflammasome in LS14 preadipocytes mediated by ERK1/2 signaling. J. Cell. Physiol. 233, 6232–6240. 10.1002/jcp.26490 29345311

[B5] Dizon D. S. (2017). PARP inhibitors for targeted treatment in ovarian cancer. Lancet 390, 1929–1930. 10.1016/S0140-6736(17)32418-2 28916370

[B6] Fatokun A. A. Dawson V. L. Dawson T. M. (2014). Parthanatos: mitochondrial-linked mechanisms and therapeutic opportunities. Br. J. Pharmacol. 171, 2000–2016. 10.1111/bph.12416 24684389 PMC3976618

[B7] Grasl-Kraupp B. Ruttkay-Nedecky B. Koudelka H. Bukowska K. Bursch W. Schulte-Hermann R. (1995). *In situ* detection of fragmented DNA (TUNEL assay) fails to discriminate among apoptosis, necrosis, and autolytic cell death: a cautionary note. Hepatology 21, 1465–1468. 10.1002/hep.1840210534 7737654

[B8] Himori N. Kunikata H. Shiga Y. Omodaka K. Maruyama K. Takahashi H. (2016). The association between systemic oxidative stress and ocular blood flow in patients with normal-tension glaucoma. Graefes Arch. Clin. Exp. Ophthalmol. 254, 333–341. 10.1007/s00417-015-3203-z 26514963

[B9] Hottiger M. O. Hassa P. O. Luscher B. Schuler H. Koch-Nolte F. (2010). Toward a unified nomenclature for mammalian ADP-ribosyltransferases. Trends biochem. Sci. 35, 208–219. 10.1016/j.tibs.2009.12.003 20106667

[B10] Huang H. Chen H. W. Evankovich J. Yan W. Rosborough B. R. Nace G. W. (2013). Histones activate the NLRP3 inflammasome in kupffer cells during sterile inflammatory liver injury. J. Immunol. 191, 2665–2679. 10.4049/jimmunol.1202733 23904166 PMC3777242

[B11] Ikejima M. Noguchi S. Yamashita R. Ogura T. Sugimura T. Gill D. M. (1990). The zinc fingers of human poly(ADP-ribose) polymerase are differentially required for the recognition of DNA breaks and nicks and the consequent enzyme activation. other structures recognize intact DNA. J. Biol. Chem. 265, 21907–21913. 10.1016/s0021-9258(18)45824-3 2123876

[B12] Jonas J. B. Aung T. Bourne R. R. Bron A. M. Ritch R. Panda-Jonas S. (2017). Glaucoma. Lancet 390, 2183–2193. 10.1016/S0140-6736(17)31469-1 28577860

[B13] Kadam A. Jubin T. Roychowdhury R. Begum R. (2020). Role of PARP-1 in mitochondrial homeostasis. Biochim. Biophys. Acta. Gen. Subj. 1864, 129669. 10.1016/j.bbagen.2020.129669 32553688

[B14] Kondkar A. A. Azad T. A. Sultan T. Osman E. A. Almobarak F. A. Al-Obeidan S. A. (2020). Elevated plasma level of 8-Hydroxy-2'-deoxyguanosine is associated with primary open-angle glaucoma. J. Ophthalmol. 2020, 6571413. 10.1155/2020/6571413 32411433 PMC7201519

[B15] Kovacs K. Vaczy A. Fekete K. Kovari P. Atlasz T. Reglodi D. (2019). PARP inhibitor protects against chronic hypoxia/reoxygenation-induced retinal injury by regulation of MAPKs, HIF1α, Nrf2, and NFκB. Invest. Ophthalmol. Vis. Sci. 60, 1478–1490. 10.1167/iovs.18-25936 30973576

[B16] Lanzi C. Lucarini L. Durante M. Sgambellone S. Pini A. Catarinicchia S. (2019). Role of histamine H₃ receptor antagonists on intraocular pressure reduction in rabbit models of transient ocular hypertension and glaucoma. Int. J. Mol. Sci. 20, 981. 10.3390/ijms20040981 PMC6412827 30813468

[B17] Lee Y. Karuppagounder S. S. Shin J. H. Lee Y. I. Ko H. S. Swing D. (2015). Corrigendum: parthanatos mediates AIMP2-activated age-dependent dopaminergic neuronal loss. Nat. Neurosci. 18, 1861. 10.1038/nn1215-1861a 26605883

[B18] Lee Y. Karuppagounder S. S. Shin J. H. Lee Y. I. Ko H. S. Swing D. (2013). Parthanatos mediates AIMP2-activated age-dependent dopaminergic neuronal loss. Nat. Neurosci. 16, 1392–1400. 10.1038/nn.3500 23974709 PMC3785563

[B19] Li Y. Yang Y. Zhao Y. Zhang J. Liu B. Jiao S. (2019). Astragaloside IV reduces neuronal apoptosis and parthanatos in ischemic injury by preserving mitochondrial hexokinase-II. Free Radic. Biol. Med. 131, 251–263. 10.1016/j.freeradbiomed.2018.11.033 30502455

[B20] Manganelli V. Matarrese P. Antonioli M. Gambardella L. Vescovo T. Gretzmeier C. (2020). Raft-like lipid microdomains drive autophagy initiation via AMBRA1-ERLIN1 molecular association within MAMs. Autophagy 17, 2528–2548. 10.1080/15548627.2020.1834207 33034545 PMC8496542

[B21] Misawa T. Takahama M. Kozaki T. Lee H. Zou J. Saitoh T. (2013). Microtubule-driven spatial arrangement of mitochondria promotes activation of the NLRP3 inflammasome. Nat. Immunol. 14, 454–460. 10.1038/ni.2550 23502856

[B22] Missiroli S. Patergnani S. Caroccia N. Pedriali G. Perrone M. Previati M. (2018). Mitochondria-associated membranes (MAMs) and inflammation. Cell Death Dis. 9, 329. 10.1038/s41419-017-0027-2 29491386 PMC5832426

[B23] Murakami T. Ockinger J. Yu J. Byles V. Mccoll A. Hofer A. M. (2012). Critical role for calcium mobilization in activation of the NLRP3 inflammasome. Proc. Natl. Acad. Sci. U. S. A. 109, 11282–11287. 10.1073/pnas.1117765109 22733741 PMC3396518

[B24] Narne P. Pandey V. Phanithi P. B. (2017). Interplay between mitochondrial metabolism and oxidative stress in ischemic stroke: an epigenetic connection. Mol. Cell. Neurosci. 82, 176–194. 10.1016/j.mcn.2017.05.008 28552342

[B25] Ni B. Pei W. Qu Y. Zhang R. Chu X. Wang Y. (2021). MCC950, the NLRP3 inhibitor, protects against cartilage degradation in a mouse model of osteoarthritis. Oxid. Med. Cell. Longev. 2021, 4139048. 10.1155/2021/4139048 34777685 PMC8580635

[B26] Nita M. Grzybowski A. (2016). The role of the reactive oxygen species and oxidative stress in the pathomechanism of the age-related ocular diseases and other pathologies of the anterior and posterior eye segments in adults. Oxid. Med. Cell Longev. 2016, 3164734. 10.1155/2016/3164734 26881021 PMC4736974

[B27] Park W. Chawla A. O'reilly E. M. (2021). Pancreatic cancer: a review. JAMA 326, 851–862. 10.1001/jama.2021.13027 34547082 PMC9363152

[B28] Perez-Leanos C. A. Romero-Campos H. E. Dupont G. Gonzalez-Velez V. (2021). Reduction of ER-mitochondria distance: a key feature in Alzheimer's and Parkinson's disease, and during cancer treatment. Annu. Int. Conf. IEEE Eng. Med. Biol. Soc. 2021, 4412–4415. 10.1109/EMBC46164.2021.9631090 34892198

[B29] Pronin A. Pham D. An W. Dvoriantchikova G. Reshetnikova G. Qiao J. (2019). Inflammasome activation induces pyroptosis in the retina exposed to ocular hypertension injury. Front. Mol. Neurosci. 12, 36. 10.3389/fnmol.2019.00036 30930743 PMC6425693

[B30] Qin Q. Yu N. Gu Y. Ke W. Zhang Q. Liu X. (2022). Inhibiting multiple forms of cell death optimizes ganglion cells survival after retinal ischemia reperfusion injury. Cell Death Dis. 13, 507. 10.1038/s41419-022-04911-9 35637215 PMC9151775

[B31] Rebello R. J. Oing C. Knudsen K. E. Loeb S. Johnson D. C. Reiter R. E. (2021). Prostate cancer. Nat. Rev. Dis. Prim. 7, 9. 10.1038/s41572-020-00243-0 33542230

[B32] Rosado M. M. Bennici E. Novelli F. Pioli C. (2013). Beyond DNA repair, the immunological role of PARP-1 and its siblings. Immunology 139, 428–437. 10.1111/imm.12099 23489378 PMC3719060

[B33] Sahaboglu A. Barth M. Secer E. Amo E. M. Urtti A. Arsenijevic Y. (2016). Olaparib significantly delays photoreceptor loss in a model for hereditary retinal degeneration. Sci. Rep. 6, 39537. 10.1038/srep39537 28004814 PMC5177898

[B34] Sappington R. M. Carlson B. J. Crish S. D. Calkins D. J. (2010). The microbead occlusion model: a paradigm for induced ocular hypertension in rats and mice. Invest. Ophthalmol. Vis. Sci. 51, 207–216. 10.1167/iovs.09-3947 19850836 PMC2869054

[B35] Seigel G. M. (2014). Review: R28 retinal precursor cells: the first 20 years. Mol. Vis. 20, 301–306. 24644404 PMC3955414

[B36] Sethi G. S. Sharma S. Naura A. S. (2019). PARP inhibition by olaparib alleviates chronic asthma-associated remodeling features via modulating inflammasome signaling in mice. IUBMB Life 71, 1003–1013. 10.1002/iub.2048 30964965

[B37] Su Y. Huang X. Huang Z. Huang T. Xu Y. Yi C. (2020). STAT3 localizes in mitochondria-associated ER membranes instead of in mitochondria. Front. Cell Dev. Biol. 8, 274. 10.3389/fcell.2020.00274 32391361 PMC7188950

[B38] Tezel G. Yang X. Luo C. Peng Y. Sun S. L. Sun D. (2007). Mechanisms of immune system activation in glaucoma: oxidative stress-stimulated antigen presentation by the retina and optic nerve head glia. Invest. Ophthalmol. Vis. Sci. 48, 705–714. 10.1167/iovs.06-0810 17251469 PMC2494942

[B39] Yao K. Zhao Y. Jin P. Lou X. Luo Z. Zhang H. (2021). Involvement of the NLRC4 inflammasome in promoting retinal ganglion cell death in an acute glaucoma mouse model. Exp. Eye Res. 203, 108388. 10.1016/j.exer.2020.108388 33333046

[B40] Yuan L. Liu Q. Wang Z. Hou J. Xu P. (2020). EI24 tethers endoplasmic reticulum and mitochondria to regulate autophagy flux. Cell. Mol. Life Sci. 77, 1591–1606. 10.1007/s00018-019-03236-9 31332481 PMC11104867

[B41] Zhou R. Yazdi A. S. Menu P. Tschopp J. (2011). A role for mitochondria in NLRP3 inflammasome activation. Nature 469, 221–225. 10.1038/nature09663 21124315

