# Supplementary figures and images for: Olaparib, a PARP-1 inhibitor, protects retinal cells from ocular hypertension-associated oxidative damage
Source: Front Cell Dev Biol. 2022 Aug 26;10:925835. doi: 10.3389/fcell.2022.925835 (PMC9459396; doi:10.3389/fcell.2022.925835)

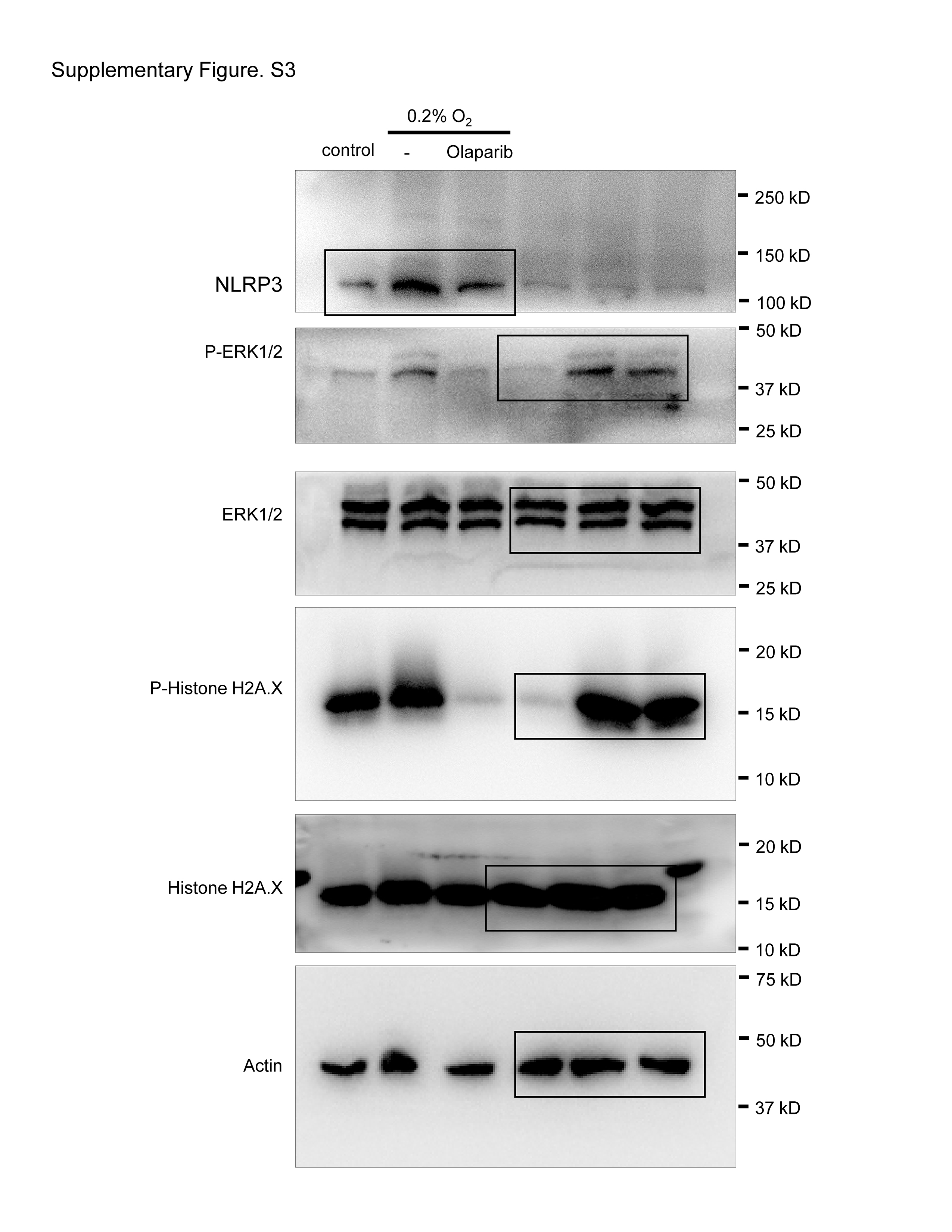

Supplement: Supplementary file 2 [file Image3.jpg]

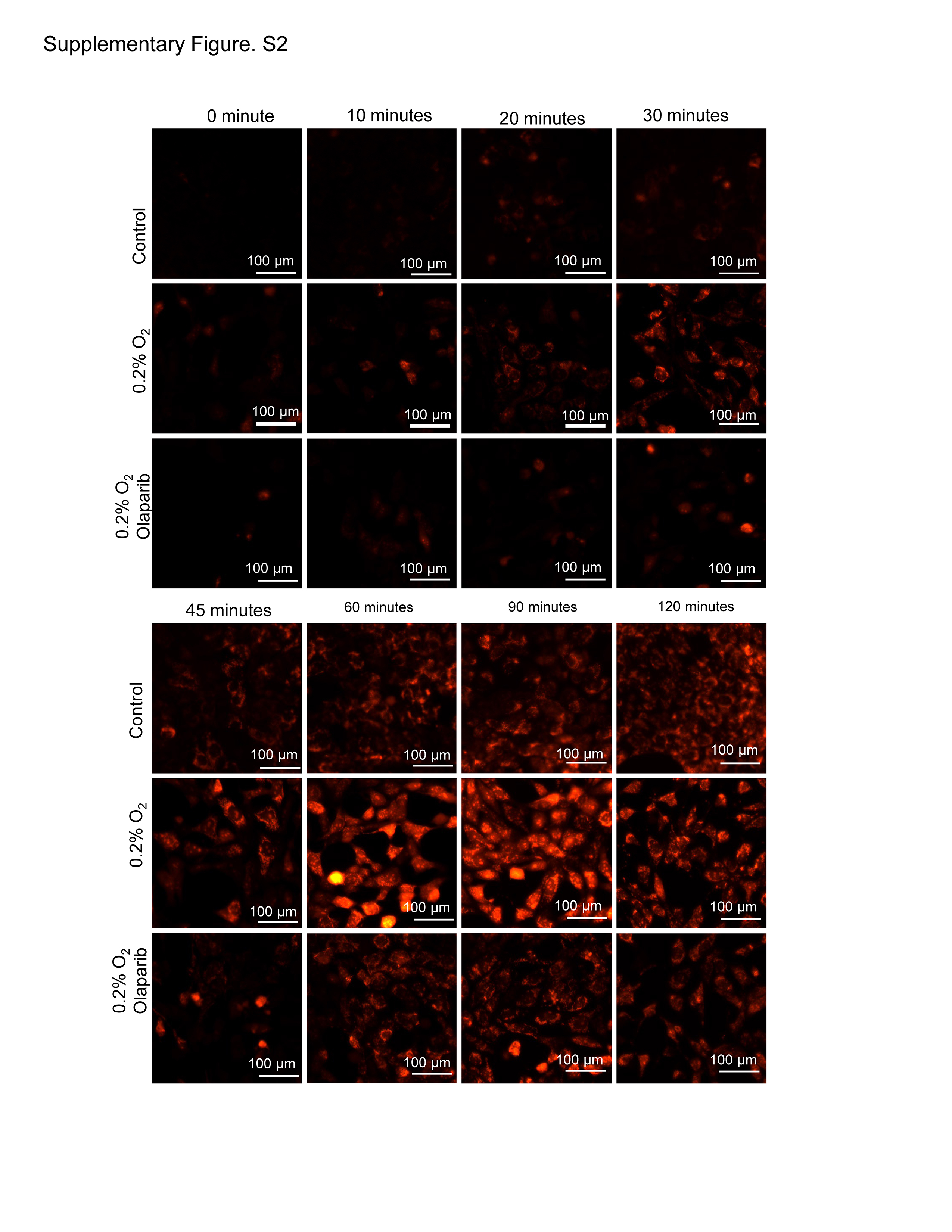

Supplement: Supplementary file 3 [file Image2.jpg]

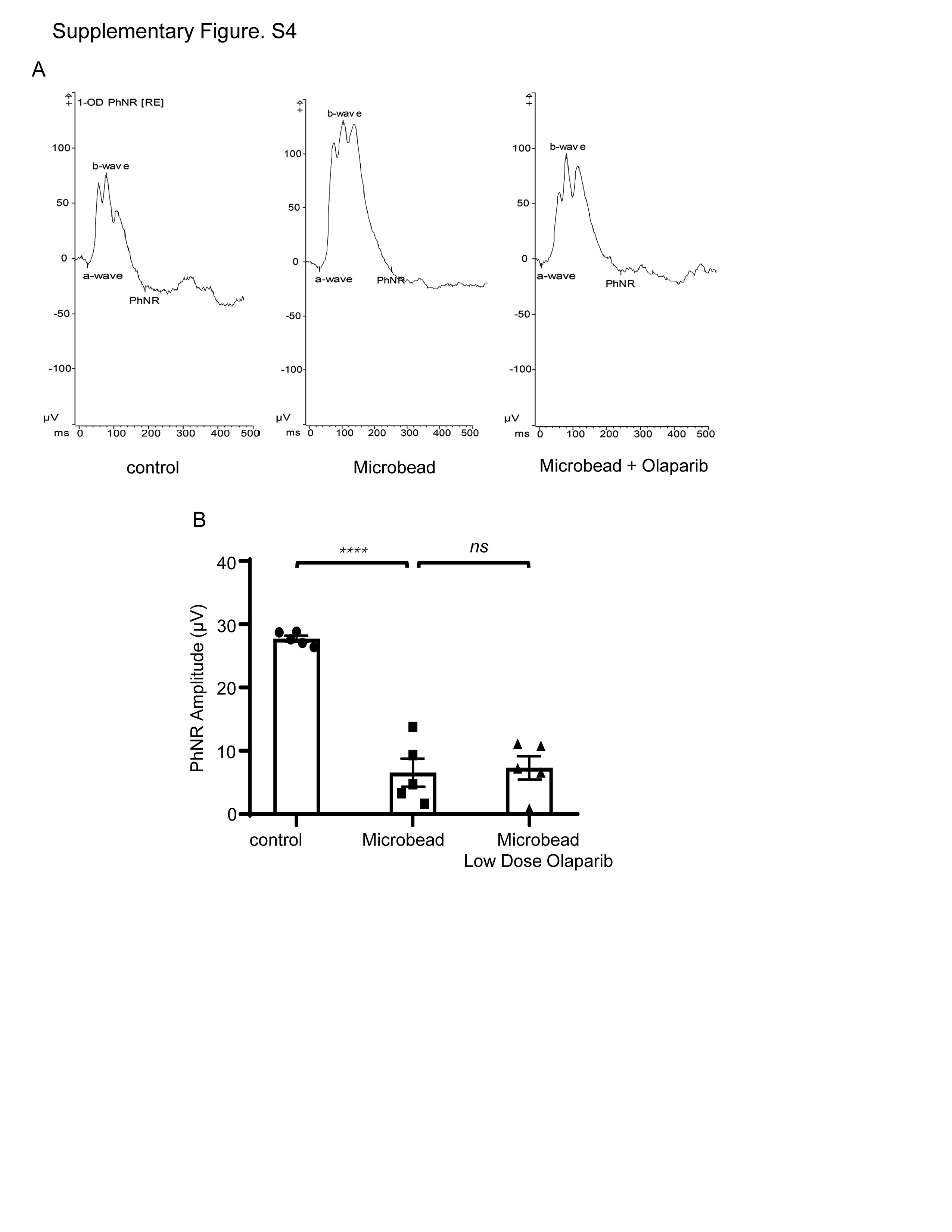

Supplement: Supplementary file 4 [file Image4.jpg]

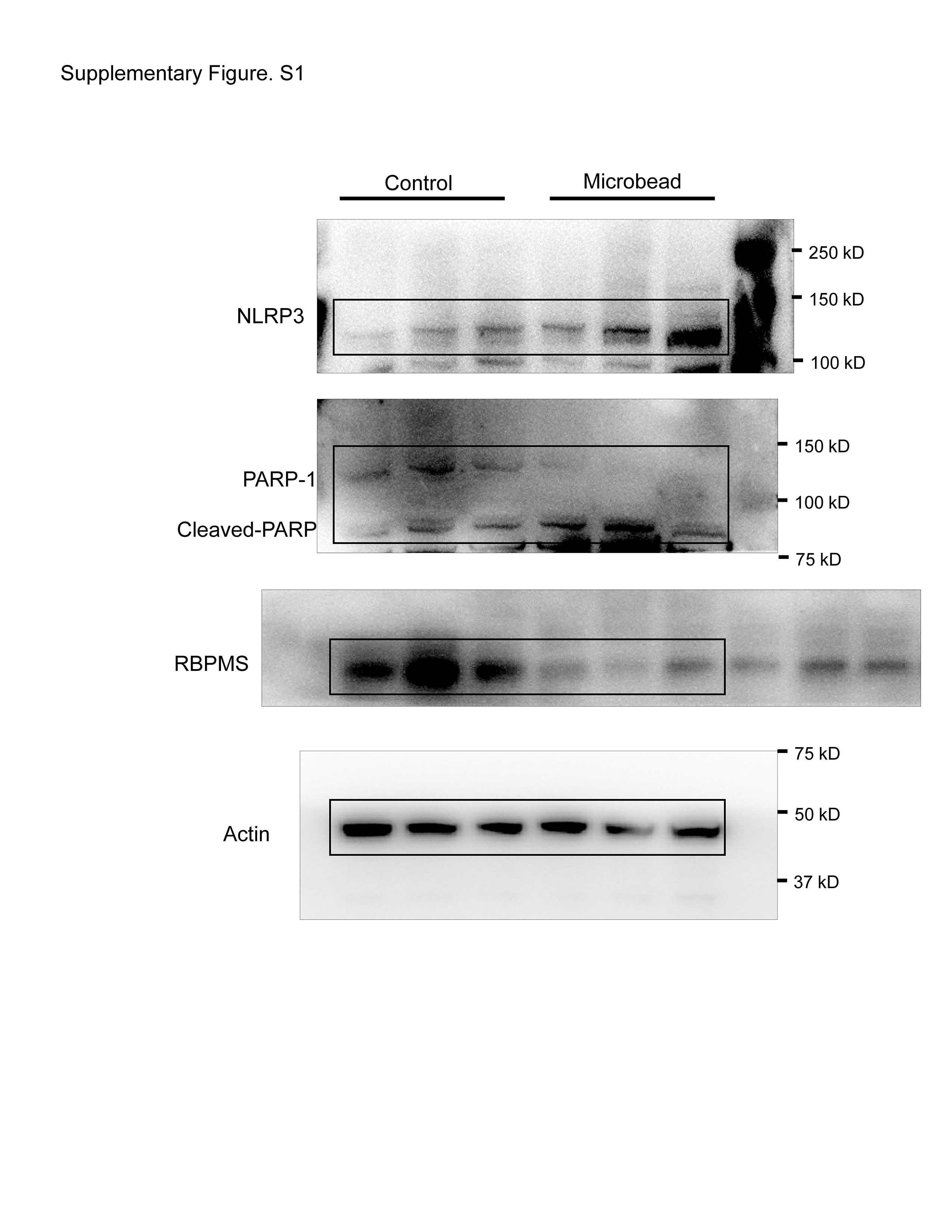

Supplement: Supplementary file 5 [file Image1.jpg]
