# Supplementary material for: Olaparib, a PARP-1 inhibitor, protects retinal cells from ocular hypertension-associated oxidative damage
Source: Front Cell Dev Biol. 2022 Aug 26;10:925835. doi: 10.3389/fcell.2022.925835 (PMC9459396; doi:10.3389/fcell.2022.925835)
Supplement: Supplementary file 6 [file DataSheet1.docx]

## Supplementary Figure Captions

**Supplementary Figure. 1** Original western blots for Figure. 3.

**Supplementary Figure. 2** Representative fluorescent micrographs for Rhod-2 staining in Figure. 8. Hypoxia increased mitochondrial calcium flux as early as 30 minutes, and this effect achieved a peak at 60 minutes. Olaparib (100 nM) relieved hypoxia-induced Ca^2+^ influx. Scale bars, 100 μm.

**Supplementary Figure. 3** Original western blots for Figure. 9.

**Supplementary Figure. 4** Low dose Olaparib failed to rescue the PhNR in the COH rats. (**A**) Typical photopic negative responses (PhNR) responses in control, Microbead and Microbead + Low dose Olaparib group. **(B)** Statistical analysis of PhNR amplitudes in the above groups. Data are presented as the mean ± SEM (n = 5 rats per group). *P*-value was determined using one-way ANOVA with Tukey’s test for multiple comparisons. * *P* < 0.0001.

**Supplementary Figure. 5** Timeline for animal experiment.
